# A91 PYOGENIC LIVER ABSCESSES DUE TO FUSOBACTERIUM NUCLEATUM ARE ASSOCIATED WITH SEVERE INFLAMMATORY RESPONSE AND ACUTE LIVER FAILURE: A SYSTEMATIC REVIEW AND META-ANALYSIS

**DOI:** 10.1093/jcag/gwad061.091

**Published:** 2024-02-14

**Authors:** N Hossein-Javaheri, M Cloutier, C Dennis, M Ramesh, I Singh, A Aijaz, H Gohil, C J Miranda

**Affiliations:** Internal Medicine, University at Buffalo, Buffalo, NY; Internal Medicine, University at Buffalo, Buffalo, NY; Internal Medicine, University at Buffalo, Buffalo, NY; Internal Medicine, University at Buffalo, Buffalo, NY; Internal Medicine, University at Buffalo, Buffalo, NY; Internal Medicine, University at Buffalo, Buffalo, NY; Internal Medicine, University at Buffalo, Buffalo, NY; Gastroenterology, University at Buffalo, Buffalo, NY

## Abstract

**Background:**

Pyogenic liver abscesses (PLAs) are uncommon entities with potentially devastating consequences requiring early diagnosis and treatment. *Fusobacterium nucleatum* is an anaerobic, gram-negative bacterium that is a rare cause of liver abscesses. *F. nucleatum* initiates a severe pro-inflammatory cascade that promotes abscess formation through the gut-liver axis especially when the gastrointestinal (GI) barrier is compromised.

**Aims:**

In this study, we explore the characteristics of PLAs due to *F.nucleatum*, including the duration and mode of therapy. This may inform clinical decisions when treating persons with suspected *F.nucleatum* PLAs and improve mortality.

**Methods:**

A systematic literature search was conducted in MEDLINE, PubMed, and the Cochrane Library, from 1977 to September 2023. We included full-text abstracts and articles written in English that reported patients with *F.nucleatum* PLA aged ampersand:003E18. Demographics, history of GI diseases, pertinent laboratory markers, abscess characteristics, and the approaches taken to treat the abscess were collected. Pooled data were assessed qualitatively and reported as mean±SEM when appropriate.

**Results:**

We included 32 studies. The mean patient age was 52±20 years with 71% being male. In total, 47% of patients had a recent history of GI disease: sigmoid diverticulosis (n=6), colonic adenomas (n=5), duodenitis (n=2), appendicitis (n=1) and ulcerative colitis (n=1). On presentation, only 53% (n=17) reported abdominal pain. Labs were significant for leukocytosis at 22±2 K/µL, C-reactive protein 199±37 mg/L, aspartate transferase (AST) 128±24 U/L, alanine aminotransferase (ALT) 116±16 U/L, Alkaline phosphatase (ALP) 275±35 IU/L, and bilirubin 11±3 mg/dL suggestive of severe inflammatory response and cholestatic liver injury. The average abscess size was approximately 10.2±0.7 cm, 28% of which had loculations (n=9) requiring multiple drainages (n=7) or laparoscopy (n=2). The initial antibiotic of choice was piperacillin/tazobactam (Zosyn) (n=12), ceftriaxone + metronidazole (n=7), Ampicillin/Sulbactam (Unasyn) (n=5), followed by Meropenem, Metronidazole, and Ciprofloxacin. The estimated length of stay was 23±4 days with a need for long-term antibiotic therapy (54±10 days) upon discharge. The majority of patients were discharged on Metronidazole (n=11), or Amoxicillin/Clavulanic acid (Augmentin) (n=6). With appropriate source control, the estimated survival was 91% (n=29).

**Conclusions:**

Our findings suggest that PLAs due to *F.nucleatum* are associated with large abscesses, a severe inflammatory response, cholestatic liver injury, and prolonged hospitalization. Multiple drainages with targeted gram-negative coverage may be required for adequate source control and favorable outcomes.

**Funding Agencies:**

None

